# Crystal structure of *catena*-poly[[aqua­bis­(4-cyano­benzoato-κ*O*)copper(II)]-μ-*N*,*N*-di­ethyl­nicotinamide-κ^2^ *N* ^1^:*O*]

**DOI:** 10.1107/S205698901601183X

**Published:** 2016-07-22

**Authors:** Nurcan Akduran, Hacali Necefoğlu, Ömer Aydoğdu, Tuncer Hökelek

**Affiliations:** a SANAEM, Saray Mahallesi, Atom Caddesi, No. 27, 06980 Saray-Kazan, Ankara, Turkey; b Department of Chemistry, Kafkas University, 36100 Kars, Turkey; c International Scientific Research Centre, Baku State University, 1148 Baku, Azerbaijan; d Department of Physics, Hacettepe University, 06800 Beytepe, Ankara, Turkey

**Keywords:** crystal structure, coordination polymers, copper(II) benzoates, nicotinamide ligands

## Abstract

The asymmetric unit of the title polymeric compound, [Cu(C_8_H_4_NO_2_)_2_(C_10_H_14_N_2_O)(H_2_O)]_*n*_, contains one Cu^II^ atom, one coordinating water mol­ecule, two 4-cyano­benzoate (CB) ligands and one coordinating *N*,*N*-di­ethyl­nicotinamide (DENA) mol­ecule. The DENA ligand acts as a bis-monodentate ligand, while the CB anions are monodentate. Two O atoms of the CB ligands, one O atom of the water mol­ecule and the pyridine N atom of the DENA ligand form a slightly distorted square-planar arrangement around the Cu^II^ atom which is completed to a square-pyramidal coordination by the apically placed O atom of the DENA ligand, with a Cu—O distance of 2.4303 (15) Å. In the two CB anions, the carboxyl­ate groups are twisted relative to the attached benzene rings by 2.19 (12) and 3.87 (15)°, while the benzene rings are oriented at a dihedral angle of 5.52 (8)°. The DENA ligands bridge adjacent Cu^2+^ ions, forming polymeric coordination chains running along the *b* axis. In the crystal, strong water–carboxyl­ate O—H⋯O hydrogen bonds link adjacent chains into layers parallel to (10-1) and weak C—H⋯O hydrogen bonds further stabilize the crystal structure. The cyano group C and N atoms of one of the CB ligands are disordered over two sets of sites with equal occupancies.

## Abstract 2

The asymmetric unit of the title polymeric compound contains one Cu^II^ atom, one coordinating water mol­ecule, two 4-cyano­benzoate (CB) ligands and one coordinating *N*,*N*-di­ethyl­nicotinamide (DENA) mol­ecule. The DENA ligands bridge adjacent Cu^2+^ ions, forming polymeric coordination chains running along the *b* axis.

## Chemical context   

Nicotinamide (NA) is one form of niacin. A deficiency of this vitamin leads to loss of copper from the body, known as pellagra disease. Victims of pellagra show unusually high serum and urinary copper levels (Krishnamachari, 1974 ▸). The nicotinic acid derivative *N*,*N*-di­ethyl­nicotinamide (DENA) is an important respiratory stimulant (Bigoli *et al.*, 1972 ▸). The structures of some complexes obtained from the reactions of transition metal(II) ions with NA and DENA as ligands, *e.g*. [Ni(NA)_2_(C_7_H_4_ClO_2_)_2_(H_2_O)_2_] (Hökelek *et al.*, 2009*a* ▸) and [Ni(C_7_H_4_ClO_2_)_2_(C_10_H_14_N_2_O)_2_(H_2_O)_2_] (Hökelek *et al.*, 2009*b* ▸), have been the subject of much inter­est in our laboratory. Aqua complexes of Cu^II^ benzoates containing nicotinamide or *N*-methyl­nicotinamide ligands have been studied *e.g*. [Cu(4-NO_2_bz)_2_(mna)_2_(H_2_O)] and [Cu(3,5-(NO_2_)_2_bz)_2_(NA)_2_(H_2_O)] (4-NO_2_bz = 4-nitro­benzoate, mna = *N*-methyl­nicotinamide, 3,5-(NO_2_)_2_bz = 3,5-di­nitro­benzoate) (Vasková *et al.*, 2014 ▸) and [Cu_2_(C_8_H_7_O_3_)_4_(C_6_H_6_N_2_O)_2_(H_2_O)_2_] (Hökelek *et al.*, 2010 ▸). To the best of our knowledge, the title compound is the first polymeric copper compound with a similar set of ligands.
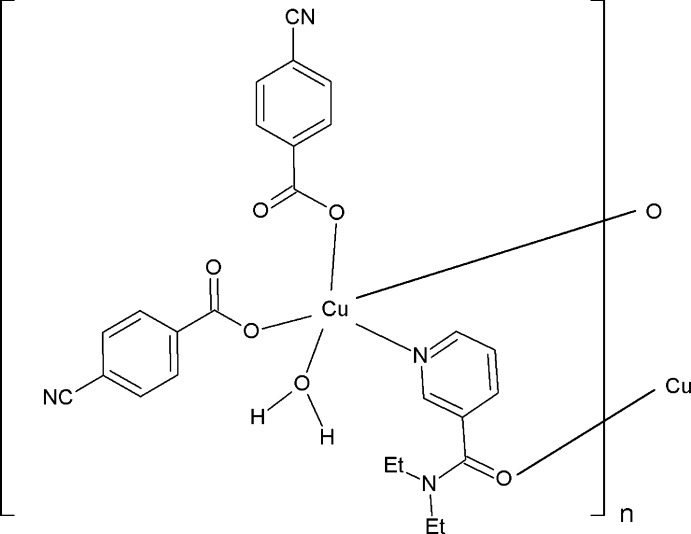


Transition metal complexes with biochemical mol­ecules show inter­esting physical and/or chemical properties, through which they may find applications in biological systems (Antolini *et al.*, 1982 ▸). Some benzoic acid derivatives, such as 4-amino­benzoic acid, have been extensively reported in coordination chemistry, as bifunctional organic ligands, due to the varieties of their coordination modes (Chen & Chen, 2002 ▸; Amiraslanov *et al.*, 1979 ▸; Hauptmann *et al.*, 2000 ▸).

The structure–function–coordination relationships of the aryl­carboxyl­ate ion in Cu^II^ complexes of benzoic acid derivatives may change depending on the nature and position of the substituent on the benzene ring, the nature of the additional ligand mol­ecule or solvent, and the pH and temperature of synthesis (Shnulin *et al.*, 1981 ▸; Nadzhafov *et al.*, 1981 ▸; Antsyshkina *et al.*, 1980 ▸; Adiwidjaja *et al.*, 1978 ▸). When pyridine and its derivatives are used instead of water mol­ecules, the structure is completely different (Catterick *et al.*, 1974 ▸). In this context, we synthesized a Cu^II^-containing compound with 4-cyano­benzoate (CB) and DENA ligands, namely *catena*-poly[[aqua­bis­(4-cyano­benzoato-κ*O*)copper(II)]-μ-*N*,*N*-diethyl­nicotinamide-κ^2^ *N* ^1^:*O*], [Cu(DENA)(CB)_2_(H_2_O)]_*n*_, and report herein its crystal structure.

## Structural commentary   

**Figure 1:**
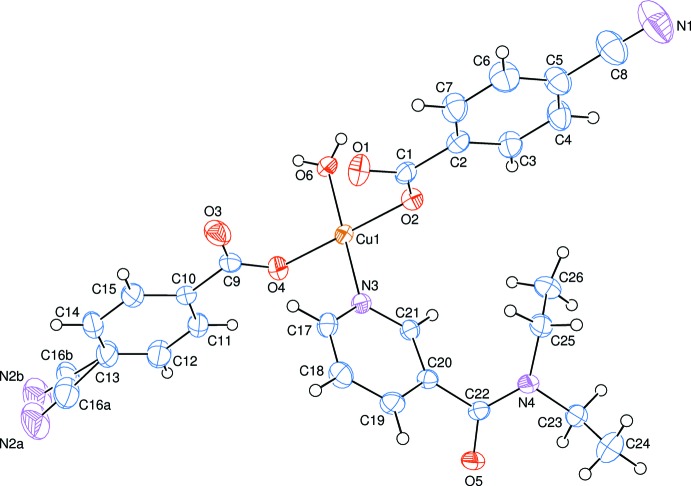
The asymmetric unit of the title mol­ecule with the atom-numbering scheme. Displacement ellipsoids are drawn at the 50% probability level.

**Figure 2:**
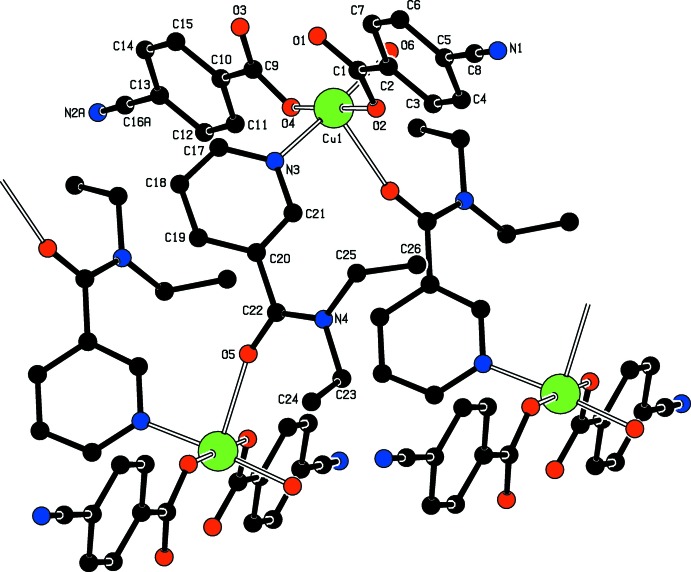
Part of the polymeric chain in the title compound. One part of the disordered CN group and H atoms have been omitted for clarity.

The asymmetric unit of the title polymeric compound contains one Cu^II^ atom, one coordinating water mol­ecule, two 4-cyano­benzoate (CB) anions and one *N*,*N*-di­ethyl­nicotinamide (DENA) ligand; the DENA ligand acts as a bis-monodentate ligand, while the CB anions are monodentate (Fig. 1 ▸). The DENA ligands bridge adjacent Cu^II^ ions, forming polymeric chains (Fig. 2 ▸) running along the *b* axis.

The two carboxyl­ate O atoms (O2 and O4) of the CB anions, the coordinating water O atom (O6) and the N atom (N3) of the DENA ligand form a slightly distorted square-planar arrangement around the Cu atom, while the distorted square-pyramidal coordination is completed by the O atom (O5) of the DENA ligand at a distance of 2.4303 (15) Å (Table 1 ▸ and Fig. 2 ▸). A more remote O atom at 2.8500 (15) Å defines a tetragonally distorted CuNO_3+2_ octahedron.

In the carboxyl­ate groups, the C—O bonds for coordinating O atoms are 0.028 (3) Å {for C1—O1 [1.244 (3) Å] and C1—O2 [1.272 (3) Å]} and 0.041 (3) Å {for C9—O3 [1.232 (3) Å] and C9—O4 [1.273 (3) Å]} longer than those of the non-coordinating ones, in which they indicate delocalized bonding arrangements rather than localized single and double bonds.

The Cu1 atom lies −0.0054 (2) and −0.1184 (2) Å, respectively, out of the planes of the O1/O2/C1 and O3/O4/C9 carboxyl­ate groups. The O1—Cu1—O2 angle is 51.12 (6)°. The corresponding O—*M*—O (where *M* is a metal) angles are 59.76 (5) and 55.08 (5)° in [Cu(C_7_H_4_O_2_Cl)_2_(C_6_H_6_N_2_O)_2_] (Bozkurt *et al.*, 2013 ▸), 53.50 (14)° in [Cu_2_(C_8_H_5_O_3_)_4_(C_6_H_6_N_2_O)_4_] (Sertçelik *et al.*, 2013 ▸), 57.75 (2)° in [Cu(C_7_H_4_FO_2_)_2_(C_7_H_5_FO_2_)(C_6_H_6_N_2_O)_2_] (Necefoğlu *et al.*, 2011 ▸) and 55.2 (1)° in [Cu(Asp)_2_(py)_2_] (where Asp is acetyl­salicylate and py is pyridine) (Greenaway *et al.*, 1984 ▸).

The dihedral angles between the carboxyl­ate groups [(O1/O2/C1) and (O3/O4/C9)] and the adjacent benzene rings [*A* (C2–C7) and *B* (C10–C15)] are 2.19 (12) and 3.87 (15)°, respectively, while the benzene and pyridine [*C* (N3/C17–C21)] rings are oriented at dihedral angles of *A*/*B* = 5.52 (8), *A*/*C* = 88.66 (7) and *B*/*C* = 85.85 (7)°.

## Supra­molecular features   

In the crystal, strong O—H_water_ ⋯ O_carboxyl­ate_ hydrogen bonds (Table 2 ▸) link adjacent chains into layers parallel to (10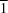). Weak inter­molecular C—H_DENA_ ⋯ O_carboxyl­ate_, C—H_DENA_ ⋯ O_DENA_ and C—H_DENA_ ⋯ O_water_ hydrogen bonds (Table 2 ▸) may further stabilize the crystal structure.

## Synthesis and crystallization   

The title compound was prepared by the reaction of CuSO_4_·5H_2_O (1.24 g, 5 mmol) in H_2_O (50 ml) and di­ethyl­nicotinamide (1.78 g, 10 mmol) in H_2_O (10 ml) with sodium 4-cyano­benzoate (1.69 g, 10 mmol) in H_2_O (100 ml). The mixture was filtered and set aside to crystallize at ambient temperature for several days, giving translucent dark-blue single crystals.

## Refinement   

The experimental details including the crystal data, data collection and refinement are summarized in Table 3 ▸. Atoms H61 and H62 (for H_2_O) were located in a difference Fourier map and were refined by applying restrains [O—H = 0.85 (2) Å]. The C-bound H atoms were positioned geometrically with C—H = 0.93, 0.97 and 0.96 Å, for aromatic, methyl­ene and methyl H-atoms, respectively, and constrained to ride on their parent atoms, with *U*_iso_(H) = *k* × *U*_eq_(C), where *k* = 1.5 for methyl H atoms and *k* = 1.2 for aromatic and methyl­ene H atoms. The CN substituents of one of the benzoate ligands are disordered over two sets of sites with equal occupancies.

## Supplementary Material

Crystal structure: contains datablock(s) I, global. DOI: 10.1107/S205698901601183X/gk2662sup1.cif

Structure factors: contains datablock(s) I. DOI: 10.1107/S205698901601183X/gk2662Isup2.hkl

CCDC reference: 1494903

Additional supporting information: crystallographic information; 3D view; checkCIF report

## Figures and Tables

**Table 1:** Selected geometric parameters (Å, °)

Cu1—O1	2.8500 (15)	Cu1—O5^i^	2.4303 (15)
Cu1—O2	1.9595 (14)	Cu1—N3	1.9999 (16)
Cu1—O4	1.9400 (14)	O6—Cu1	1.9503 (15)
			
O2—Cu1—O5^i^	90.12 (6)	O6—Cu1—O2	88.67 (6)
O2—Cu1—N3	89.16 (6)	O6—Cu1—O5^i^	93.94 (6)
O4—Cu1—O5^i^	90.54 (6)	O6—Cu1—N3	175.09 (7)
O4—Cu1—O6	91.40 (7)	N3—Cu1—O5^i^	90.47 (6)
O4—Cu1—N3	90.73 (6)		

Symmetry code: (i) 
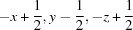
.

**Table 2:** Hydrogen-bond geometry (Å, °)

*D*—H⋯*A*	*D*—H	H⋯*A*	*D*⋯*A*	*D*—H⋯*A*
O6—H61⋯O3^ii^	0.81 (2)	1.83 (2)	2.630 (2)	171 (3)
O6—H62⋯O1^ii^	0.79 (2)	1.90 (2)	2.673 (2)	166 (3)
C18—H18⋯O2^iii^	0.93	2.55	3.460 (3)	166
C21—H21⋯O5^i^	0.93	2.45	3.054 (3)	123
C23—H23*B*⋯O6^iv^	0.97	2.32	3.208 (3)	152

Symmetry codes: (i) 
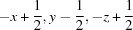
; (ii) 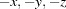; (iii) 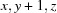; (iv) 
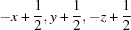
.

**Table 3:** Experimental details

Crystal data	
Chemical formula	[Cu(C_8_H_4_NO_2_)_2_(C_10_H_14_N_2_O)(H_2_O)]
*M*_r_	552.04
Crystal system, space group	Monoclinic, *P*2_1_/*n*
Temperature (K)	296
*a*, *b*, *c* (Å)	14.6207 (4), 8.0160 (3), 22.2892 (5)
β (°)	101.725 (3)
*V* (Å^3^)	2557.78 (13)
*Z*	4
Radiation type	Mo *K*α
μ (mm^−1^)	0.90
Crystal size (mm)	0.45 × 0.36 × 0.11

Data collection	
Diffractometer	Bruker *SMART* BREEZE CCD
Absorption correction	Multi-scan (*SADABS*; Bruker, 2012 ▸)
*T*_min_, *T*_max_	0.671, 0.912
No. of measured, independent and observed [*I* > 2σ(*I*)] reflections	42530, 6403, 4871
*R*_int_	0.044
(sin θ/λ)_max_ (Å^−1^)	0.669

Refinement	
*R*[*F* ^2^ > 2σ(*F* ^2^)], *wR*(*F* ^2^), *S*	0.042, 0.103, 1.05
No. of reflections	6403
No. of parameters	362
No. of restraints	2
H-atom treatment	H atoms treated by a mixture of independent and constrained refinement
Δρ_max_, Δρ_min_ (e Å^−3^)	0.44, −0.47

Computer programs: *APEX2* and *SAINT* (Bruker, 2012 ▸), *SHELXS97* and *SHELXL97* (Sheldrick, 2008 ▸), *ORTEP-3 for Windows* and *WinGX* (Farrugia, 2012 ▸) and *PLATON* (Spek, 2009 ▸).

## supplementary crystallographic information

### Crystal data

**Table:**

[Cu(C_8_H_4_NO_2_)_2_(C_10_H_14_N_2_O)(H_2_O)]	*F*(000) = 1140
*M**_r_* = 552.04	*D*_x_ = 1.434 Mg m^−^^3^
Monoclinic, *P*2_1_/*n*	Mo *K*α radiation, λ = 0.71073 Å
Hall symbol: -P 2yn	Cell parameters from 9911 reflections
*a* = 14.6207 (4) Å	θ = 2.7–28.3°
*b* = 8.0160 (3) Å	µ = 0.90 mm^−^^1^
*c* = 22.2892 (5) Å	*T* = 296 K
β = 101.725 (3)°	Prism, translucent dark blue
*V* = 2557.78 (13) Å^3^	0.45 × 0.36 × 0.11 mm
*Z* = 4	

### Data collection

**Table:**

Bruker SMART BREEZE CCD diffractometer	6403 independent reflections
Radiation source: fine-focus sealed tube	4871 reflections with *I* > 2σ(*I*)
Graphite monochromator	*R*_int_ = 0.044
φ and ω scans	θ_max_ = 28.4°, θ_min_ = 1.5°
Absorption correction: multi-scan (SADABS; Bruker, 2012)	*h* = −19→19
*T*_min_ = 0.671, *T*_max_ = 0.912	*k* = −10→10
42530 measured reflections	*l* = −29→29

### Refinement

**Table:**

Refinement on *F*^2^	Primary atom site location: structure-invariant direct methods
Least-squares matrix: full	Secondary atom site location: difference Fourier map
*R*[*F*^2^ > 2σ(*F*^2^)] = 0.042	Hydrogen site location: inferred from neighbouring sites
*wR*(*F*^2^) = 0.103	H atoms treated by a mixture of independent and constrained refinement
*S* = 1.05	*w* = 1/[σ^2^(*F*_o_^2^) + (0.0489*P*)^2^ + 0.8094*P*] where *P* = (*F*_o_^2^ + 2*F*_c_^2^)/3
6403 reflections	(Δ/σ)_max_ < 0.001
362 parameters	Δρ_max_ = 0.44 e Å^−^^3^
2 restraints	Δρ_min_ = −0.47 e Å^−^^3^

### Special details

**Table:**

Geometry. All esds (except the esd in the dihedral angle between two l.s. planes) are estimated using the full covariance matrix. The cell esds are taken into account individually in the estimation of esds in distances, angles and torsion angles; correlations between esds in cell parameters are only used when they are defined by crystal symmetry. An approximate (isotropic) treatment of cell esds is used for estimating esds involving l.s. planes.
Refinement. Refinement of F^2^ against ALL reflections. The weighted R-factor wR and goodness of fit S are based on F^2^, conventional R-factors R are based on F, with F set to zero for negative F^2^. The threshold expression of F^2^ > 2sigma(F^2^) is used only for calculating R-factors(gt) etc. and is not relevant to the choice of reflections for refinement. R-factors based on F^2^ are statistically about twice as large as those based on F, and R- factors based on ALL data will be even larger.

### Fractional atomic coordinates and isotropic or equivalent isotropic displacement parameters (Å^2^)

**Table:**

	*x*	*y*	*z*	*U*_iso_*/*U*_eq_	Occ. (<1)
Cu1	0.090509 (15)	0.08013 (3)	0.097687 (11)	0.03134 (9)	
O1	0.16527 (10)	0.1123 (2)	−0.01029 (8)	0.0518 (4)	
O2	0.20187 (9)	−0.02498 (18)	0.07812 (7)	0.0381 (3)	
O3	−0.07154 (12)	0.2958 (2)	0.02356 (8)	0.0556 (4)	
O4	−0.01992 (9)	0.18571 (19)	0.11618 (7)	0.0444 (4)	
O5	0.37494 (11)	0.45382 (19)	0.30110 (7)	0.0472 (4)	
O6	0.01725 (10)	−0.10957 (19)	0.05986 (7)	0.0365 (3)	
H61	0.0397 (17)	−0.162 (3)	0.0354 (11)	0.063 (9)*	
H62	−0.0380 (12)	−0.108 (3)	0.0510 (12)	0.060 (8)*	
N1	0.5925 (2)	−0.3371 (4)	−0.06545 (14)	0.0950 (10)	
N2A	−0.4017 (6)	0.7313 (12)	0.1822 (4)	0.109 (3)	0.50
N2B	−0.4394 (6)	0.6248 (14)	0.1870 (5)	0.116 (3)	0.50
N3	0.16793 (10)	0.2791 (2)	0.12940 (7)	0.0314 (4)	
N4	0.43627 (11)	0.2351 (2)	0.25951 (7)	0.0358 (4)	
C1	0.21593 (13)	0.0157 (3)	0.02560 (10)	0.0376 (5)	
C2	0.29915 (14)	−0.0606 (3)	0.00607 (10)	0.0374 (5)	
C3	0.35893 (15)	−0.1668 (3)	0.04427 (11)	0.0451 (5)	
H3	0.3480	−0.1906	0.0830	0.054*	
C4	0.43431 (16)	−0.2378 (3)	0.02588 (12)	0.0532 (6)	
H4	0.4739	−0.3092	0.0520	0.064*	
C5	0.45085 (16)	−0.2027 (3)	−0.03156 (12)	0.0506 (6)	
C6	0.39130 (18)	−0.0968 (3)	−0.07040 (12)	0.0572 (7)	
H6	0.4022	−0.0733	−0.1092	0.069*	
C7	0.31624 (16)	−0.0267 (3)	−0.05161 (11)	0.0501 (6)	
H7	0.2765	0.0443	−0.0778	0.060*	
C8	0.5301 (2)	−0.2783 (4)	−0.05100 (13)	0.0676 (8)	
C9	−0.07600 (14)	0.2734 (3)	0.07759 (11)	0.0385 (5)	
C10	−0.15334 (13)	0.3569 (3)	0.10182 (10)	0.0371 (5)	
C11	−0.16399 (15)	0.3308 (3)	0.16119 (11)	0.0476 (6)	
H11	−0.1239	0.2585	0.1866	0.057*	
C12	−0.23369 (18)	0.4116 (4)	0.18294 (13)	0.0642 (8)	
H12	−0.2405	0.3949	0.2231	0.077*	
C13	−0.29347 (19)	0.5176 (4)	0.14476 (13)	0.0706 (9)	
C14	−0.2838 (2)	0.5436 (4)	0.08554 (13)	0.0692 (9)	
H14	−0.3245	0.6149	0.0600	0.083*	
C15	−0.21392 (17)	0.4638 (3)	0.06426 (11)	0.0521 (6)	
H15	−0.2070	0.4817	0.0242	0.062*	
C16A	−0.3521 (8)	0.6441 (15)	0.1651 (6)	0.073 (3)	0.50
C16B	−0.3782 (8)	0.5688 (14)	0.1706 (6)	0.081 (3)	0.50
C17	0.15321 (14)	0.4322 (3)	0.10551 (10)	0.0382 (5)	
H17	0.1037	0.4486	0.0725	0.046*	
C18	0.20800 (16)	0.5653 (3)	0.12768 (11)	0.0440 (5)	
H18	0.1959	0.6703	0.1101	0.053*	
C19	0.28183 (15)	0.5417 (3)	0.17672 (11)	0.0401 (5)	
H19	0.3193	0.6312	0.1930	0.048*	
C20	0.29939 (13)	0.3835 (2)	0.20134 (9)	0.0297 (4)	
C21	0.24061 (12)	0.2563 (2)	0.17641 (9)	0.0308 (4)	
H21	0.2516	0.1497	0.1928	0.037*	
C22	0.37445 (13)	0.3587 (3)	0.25778 (9)	0.0316 (4)	
C23	0.50883 (16)	0.2147 (3)	0.31541 (11)	0.0538 (6)	
H23A	0.5285	0.0990	0.3190	0.065*	
H23B	0.4825	0.2417	0.3508	0.065*	
C24	0.59187 (19)	0.3226 (5)	0.31558 (17)	0.0892 (11)	
H24A	0.6363	0.3072	0.3533	0.134*	
H24B	0.5727	0.4373	0.3118	0.134*	
H24C	0.6201	0.2927	0.2818	0.134*	
C25	0.44567 (16)	0.1255 (3)	0.20873 (11)	0.0484 (6)	
H25A	0.5103	0.1255	0.2042	0.058*	
H25B	0.4079	0.1690	0.1711	0.058*	
C26	0.4156 (2)	−0.0535 (3)	0.21815 (15)	0.0693 (8)	
H26A	0.4343	−0.1249	0.1882	0.104*	
H26B	0.3490	−0.0578	0.2137	0.104*	
H26C	0.4447	−0.0905	0.2585	0.104*	

### Atomic displacement parameters (Å^2^)

**Table:**

	*U*^11^	*U*^22^	*U*^33^	*U*^12^	*U*^13^	*U*^23^
Cu1	0.02943 (12)	0.03170 (15)	0.03000 (15)	0.00186 (9)	−0.00075 (9)	−0.00126 (10)
O1	0.0366 (8)	0.0593 (11)	0.0556 (11)	0.0051 (7)	0.0006 (7)	0.0137 (8)
O2	0.0365 (7)	0.0379 (8)	0.0394 (9)	0.0017 (6)	0.0066 (6)	−0.0021 (7)
O3	0.0702 (11)	0.0561 (11)	0.0428 (10)	0.0174 (9)	0.0170 (8)	−0.0003 (8)
O4	0.0369 (7)	0.0483 (9)	0.0470 (9)	0.0091 (7)	0.0059 (6)	−0.0029 (8)
O5	0.0543 (9)	0.0412 (9)	0.0399 (9)	0.0110 (7)	−0.0054 (7)	−0.0162 (7)
O6	0.0322 (7)	0.0422 (9)	0.0322 (9)	−0.0014 (6)	−0.0004 (6)	−0.0043 (7)
N1	0.103 (2)	0.105 (2)	0.091 (2)	0.0460 (19)	0.0533 (17)	0.0233 (18)
N2A	0.093 (6)	0.148 (9)	0.090 (5)	0.064 (6)	0.032 (4)	−0.015 (6)
N2B	0.099 (7)	0.158 (9)	0.098 (6)	0.068 (6)	0.036 (5)	−0.006 (7)
N3	0.0340 (8)	0.0276 (9)	0.0306 (9)	0.0021 (6)	0.0018 (6)	0.0009 (7)
N4	0.0389 (8)	0.0356 (10)	0.0299 (9)	0.0041 (7)	0.0001 (7)	−0.0068 (8)
C1	0.0310 (9)	0.0347 (11)	0.0432 (13)	−0.0045 (8)	−0.0015 (8)	−0.0038 (10)
C2	0.0353 (10)	0.0383 (12)	0.0367 (12)	−0.0047 (8)	0.0030 (8)	−0.0025 (9)
C3	0.0458 (11)	0.0527 (14)	0.0372 (13)	0.0051 (10)	0.0095 (9)	0.0072 (11)
C4	0.0512 (12)	0.0591 (16)	0.0498 (15)	0.0164 (12)	0.0118 (11)	0.0128 (13)
C5	0.0551 (13)	0.0514 (15)	0.0488 (15)	0.0079 (11)	0.0189 (11)	0.0030 (12)
C6	0.0656 (15)	0.0691 (18)	0.0408 (15)	0.0088 (13)	0.0203 (12)	0.0098 (13)
C7	0.0529 (13)	0.0542 (15)	0.0424 (14)	0.0090 (11)	0.0078 (10)	0.0116 (12)
C8	0.0784 (18)	0.070 (2)	0.0616 (18)	0.0225 (15)	0.0322 (15)	0.0129 (15)
C9	0.0379 (10)	0.0317 (11)	0.0443 (14)	−0.0021 (9)	0.0044 (9)	−0.0083 (10)
C10	0.0354 (10)	0.0354 (11)	0.0379 (13)	0.0013 (8)	0.0011 (8)	−0.0082 (10)
C11	0.0433 (11)	0.0529 (15)	0.0442 (14)	0.0105 (10)	0.0030 (9)	0.0005 (12)
C12	0.0574 (15)	0.094 (2)	0.0420 (15)	0.0211 (14)	0.0116 (11)	−0.0047 (14)
C13	0.0578 (15)	0.098 (2)	0.0520 (18)	0.0357 (16)	0.0013 (12)	−0.0187 (16)
C14	0.0707 (17)	0.075 (2)	0.0542 (18)	0.0394 (15)	−0.0058 (13)	−0.0091 (15)
C15	0.0583 (14)	0.0550 (15)	0.0394 (14)	0.0155 (12)	0.0017 (10)	−0.0026 (12)
C16A	0.063 (5)	0.107 (9)	0.051 (4)	0.027 (5)	0.015 (4)	−0.001 (6)
C16B	0.070 (7)	0.099 (9)	0.075 (6)	0.045 (5)	0.018 (5)	−0.006 (6)
C17	0.0398 (10)	0.0375 (12)	0.0346 (12)	0.0057 (9)	0.0011 (8)	0.0067 (9)
C18	0.0517 (12)	0.0284 (11)	0.0502 (15)	0.0034 (9)	0.0063 (10)	0.0109 (10)
C19	0.0459 (11)	0.0265 (11)	0.0460 (13)	−0.0051 (9)	0.0052 (9)	0.0006 (9)
C20	0.0341 (9)	0.0281 (10)	0.0271 (10)	−0.0003 (8)	0.0067 (7)	−0.0008 (8)
C21	0.0342 (9)	0.0256 (10)	0.0311 (11)	0.0018 (8)	0.0028 (7)	0.0029 (8)
C22	0.0350 (9)	0.0281 (10)	0.0305 (11)	−0.0035 (8)	0.0038 (8)	−0.0024 (9)
C23	0.0525 (13)	0.0561 (16)	0.0438 (14)	0.0200 (11)	−0.0113 (10)	−0.0138 (12)
C24	0.0473 (15)	0.101 (3)	0.108 (3)	−0.0012 (16)	−0.0109 (15)	−0.035 (2)
C25	0.0451 (12)	0.0628 (16)	0.0362 (13)	0.0142 (11)	0.0057 (9)	−0.0108 (11)
C26	0.0722 (18)	0.0439 (16)	0.083 (2)	0.0149 (13)	−0.0048 (15)	−0.0242 (14)

### Geometric parameters (Å, º)

**Table:**

Cu1—O1	2.8500 (15)	C11—C12	1.376 (3)
Cu1—O2	1.9595 (14)	C11—H11	0.9300
Cu1—O4	1.9400 (14)	C12—H12	0.9300
Cu1—O5^i^	2.4303 (15)	C13—C14	1.372 (4)
Cu1—N3	1.9999 (16)	C13—C12	1.381 (4)
O1—C1	1.244 (3)	C14—C15	1.370 (4)
O2—C1	1.272 (3)	C14—H14	0.9300
O3—C9	1.232 (3)	C15—H15	0.9300
O4—C9	1.273 (3)	C16A—N2A	1.128 (14)
O5—Cu1^ii^	2.4303 (15)	C16A—N2B	1.464 (13)
O5—C22	1.229 (2)	C16A—C13	1.458 (13)
O6—Cu1	1.9503 (15)	C16A—C16B	0.737 (13)
O6—H61	0.810 (17)	C16B—N2A	1.385 (15)
O6—H62	0.791 (17)	C16B—N2B	1.126 (13)
N2A—N2B	1.034 (11)	C16B—C13	1.525 (11)
N3—C17	1.338 (2)	C17—C18	1.365 (3)
N3—C21	1.345 (2)	C17—H17	0.9300
N4—C22	1.336 (2)	C18—C19	1.384 (3)
N4—C23	1.472 (3)	C18—H18	0.9300
N4—C25	1.461 (3)	C19—H19	0.9300
C1—C2	1.503 (3)	C20—C19	1.385 (3)
C2—C3	1.383 (3)	C21—C20	1.376 (3)
C2—C7	1.386 (3)	C21—H21	0.9300
C3—H3	0.9300	C22—C20	1.505 (3)
C4—C3	1.375 (3)	C23—C24	1.490 (4)
C4—C5	1.379 (3)	C23—H23A	0.9700
C4—H4	0.9300	C23—H23B	0.9700
C5—C6	1.387 (3)	C24—H24A	0.9600
C5—C8	1.449 (3)	C24—H24B	0.9600
C6—H6	0.9300	C24—H24C	0.9600
C7—C6	1.372 (3)	C25—C26	1.528 (4)
C7—H7	0.9300	C25—H25A	0.9700
C8—N1	1.130 (3)	C25—H25B	0.9700
C9—C10	1.506 (3)	C26—H26A	0.9600
C10—C11	1.379 (3)	C26—H26B	0.9600
C10—C15	1.385 (3)	C26—H26C	0.9600
			
O1—Cu1—O2	51.12 (6)	C14—C13—C12	120.7 (2)
O2—Cu1—O5^i^	90.12 (6)	C14—C13—C16A	112.0 (5)
O2—Cu1—N3	89.16 (6)	C14—C13—C16B	124.9 (6)
O4—Cu1—O2	179.33 (7)	C13—C14—H14	120.3
O4—Cu1—O5^i^	90.54 (6)	C15—C14—C13	119.5 (2)
O4—Cu1—O6	91.40 (7)	C15—C14—H14	120.3
O4—Cu1—N3	90.73 (6)	C10—C15—H15	119.7
O6—Cu1—O2	88.67 (6)	C14—C15—C10	120.7 (2)
O6—Cu1—O5^i^	93.94 (6)	C14—C15—H15	119.7
O6—Cu1—N3	175.09 (7)	N2A—C16A—C13	174.3 (11)
N3—Cu1—O5^i^	90.47 (6)	C13—C16A—N2B	129.6 (10)
C1—O2—Cu1	113.07 (13)	C16B—C16A—N2A	93.5 (18)
C9—O4—Cu1	123.16 (14)	C16B—C16A—N2B	48.9 (14)
C22—O5—Cu1^ii^	162.66 (14)	C16B—C16A—C13	80.8 (18)
Cu1—O6—H61	116 (2)	N2A—C16B—C13	125.1 (9)
Cu1—O6—H62	123 (2)	N2B—C16B—N2A	47.3 (7)
H61—O6—H62	112 (3)	N2B—C16B—C13	171.9 (12)
N2B—N2A—C16A	85.2 (11)	C16A—C16B—N2A	54.4 (16)
N2B—N2A—C16B	53.1 (8)	C16A—C16B—N2B	102 (2)
N2A—N2B—C16A	50.1 (8)	C16A—C16B—C13	70.7 (16)
N2A—N2B—C16B	79.6 (10)	N3—C17—C18	122.52 (19)
C17—N3—Cu1	123.86 (13)	N3—C17—H17	118.7
C17—N3—C21	118.21 (17)	C18—C17—H17	118.7
C21—N3—Cu1	117.90 (13)	C17—C18—C19	119.05 (19)
C22—N4—C23	118.27 (17)	C17—C18—H18	120.5
C22—N4—C25	126.36 (17)	C19—C18—H18	120.5
C25—N4—C23	115.08 (17)	C18—C19—C20	119.32 (19)
O1—C1—O2	124.4 (2)	C18—C19—H19	120.3
O1—C1—C2	118.6 (2)	C20—C19—H19	120.3
O2—C1—C2	117.02 (18)	C19—C20—C22	119.78 (18)
C3—C2—C7	118.8 (2)	C21—C20—C19	117.93 (18)
C3—C2—C1	121.3 (2)	C21—C20—C22	121.97 (17)
C7—C2—C1	119.89 (19)	N3—C21—C20	122.95 (18)
C2—C3—H3	119.5	N3—C21—H21	118.5
C4—C3—C2	121.1 (2)	C20—C21—H21	118.5
C4—C3—H3	119.5	O5—C22—N4	122.77 (18)
C3—C4—C5	119.6 (2)	O5—C22—C20	117.50 (17)
C3—C4—H4	120.2	N4—C22—C20	119.73 (17)
C5—C4—H4	120.2	N4—C23—C24	112.6 (2)
C4—C5—C6	119.9 (2)	N4—C23—H23A	109.1
C4—C5—C8	119.6 (2)	N4—C23—H23B	109.1
C6—C5—C8	120.5 (2)	C24—C23—H23A	109.1
C5—C6—H6	120.0	C24—C23—H23B	109.1
C7—C6—C5	120.0 (2)	H23A—C23—H23B	107.8
C7—C6—H6	120.0	C23—C24—H24A	109.5
C2—C7—H7	119.7	C23—C24—H24B	109.5
C6—C7—C2	120.6 (2)	C23—C24—H24C	109.5
C6—C7—H7	119.7	H24A—C24—H24B	109.5
N1—C8—C5	179.2 (4)	H24A—C24—H24C	109.5
O3—C9—O4	125.9 (2)	H24B—C24—H24C	109.5
O3—C9—C10	118.58 (19)	N4—C25—C26	112.5 (2)
O4—C9—C10	115.5 (2)	N4—C25—H25A	109.1
C11—C10—C9	121.09 (19)	N4—C25—H25B	109.1
C11—C10—C15	119.4 (2)	C26—C25—H25A	109.1
C15—C10—C9	119.5 (2)	C26—C25—H25B	109.1
C10—C11—H11	119.9	H25A—C25—H25B	107.8
C12—C11—C10	120.2 (2)	C25—C26—H26A	109.5
C12—C11—H11	119.9	C25—C26—H26B	109.5
C11—C12—C13	119.6 (2)	C25—C26—H26C	109.5
C11—C12—H12	120.2	H26A—C26—H26B	109.5
C13—C12—H12	120.2	H26A—C26—H26C	109.5
C12—C13—C16A	125.1 (5)	H26B—C26—H26C	109.5
C12—C13—C16B	113.2 (5)		
			
O5^i^—Cu1—O2—C1	−176.78 (14)	C9—C10—C11—C12	178.4 (2)
O6—Cu1—O2—C1	89.28 (14)	C15—C10—C11—C12	−0.5 (4)
N3—Cu1—O2—C1	−86.32 (14)	C11—C10—C15—C14	0.1 (4)
O5^i^—Cu1—O4—C9	−177.31 (16)	C9—C10—C15—C14	−178.9 (2)
O6—Cu1—O4—C9	−83.35 (16)	C10—C11—C12—C13	0.6 (4)
N3—Cu1—O4—C9	92.22 (16)	C14—C13—C12—C11	−0.2 (5)
O2—Cu1—N3—C17	117.84 (16)	C16A—C13—C12—C11	−162.1 (6)
O2—Cu1—N3—C21	−60.21 (14)	C16B—C13—C12—C11	168.1 (6)
O4—Cu1—N3—C17	−61.50 (16)	C12—C13—C14—C15	−0.2 (5)
O4—Cu1—N3—C21	120.45 (14)	C16A—C13—C14—C15	163.8 (5)
O5^i^—Cu1—N3—C17	−152.04 (16)	C16B—C13—C14—C15	−167.1 (6)
O5^i^—Cu1—N3—C21	29.90 (14)	C13—C14—C15—C10	0.3 (5)
Cu1—O2—C1—O1	0.2 (3)	N2B—C16A—N2A—C16B	3 (2)
Cu1—O2—C1—C2	−179.20 (13)	C16B—C16A—N2A—N2B	−3 (2)
Cu1—O4—C9—O3	4.2 (3)	N2A—C16A—N2B—C16B	−176 (3)
Cu1—O4—C9—C10	−175.13 (13)	C13—C16A—N2B—N2A	179.0 (17)
Cu1^ii^—O5—C22—N4	−10.6 (6)	C13—C16A—N2B—C16B	3.5 (15)
Cu1^ii^—O5—C22—C20	168.3 (4)	C16B—C16A—N2B—N2A	176 (3)
C16A—N2A—N2B—C16B	2.2 (15)	N2B—C16A—C13—C12	−76.1 (13)
C16B—N2A—N2B—C16A	−2.2 (15)	N2B—C16A—C13—C14	120.7 (11)
Cu1—N3—C17—C18	−179.16 (17)	N2B—C16A—C13—C16B	−2.6 (11)
C21—N3—C17—C18	−1.1 (3)	C16B—C16A—C13—C12	−73 (2)
Cu1—N3—C21—C20	179.03 (14)	C16B—C16A—C13—C14	123.4 (18)
C17—N3—C21—C20	0.9 (3)	N2A—C16A—C16B—N2B	3 (2)
C23—N4—C22—O5	−1.1 (3)	N2A—C16A—C16B—C13	−179.5 (11)
C23—N4—C22—C20	179.90 (19)	N2B—C16A—C16B—N2A	−3 (2)
C25—N4—C22—O5	−174.6 (2)	N2B—C16A—C16B—C13	177.3 (12)
C25—N4—C22—C20	6.5 (3)	C13—C16A—C16B—N2A	179.5 (11)
C22—N4—C23—C24	−85.3 (3)	C13—C16A—C16B—N2B	−177.3 (12)
C25—N4—C23—C24	88.8 (3)	N2B—C16B—N2A—C16A	−176 (3)
C22—N4—C25—C26	−110.6 (2)	C13—C16B—N2A—C16A	0.5 (13)
C23—N4—C25—C26	75.8 (3)	C13—C16B—N2A—N2B	176.3 (17)
O1—C1—C2—C3	179.0 (2)	C16A—C16B—N2A—N2B	176 (3)
O1—C1—C2—C7	−2.0 (3)	N2A—C16B—N2B—C16A	3 (2)
O2—C1—C2—C3	−1.6 (3)	C16A—C16B—N2B—N2A	−3 (2)
O2—C1—C2—C7	177.4 (2)	N2A—C16B—C13—C12	121.0 (11)
C1—C2—C3—C4	179.2 (2)	N2A—C16B—C13—C14	−71.3 (14)
C7—C2—C3—C4	0.2 (4)	N2A—C16B—C13—C16A	−0.4 (11)
C1—C2—C7—C6	−179.3 (2)	C16A—C16B—C13—C12	121.4 (18)
C3—C2—C7—C6	−0.2 (4)	C16A—C16B—C13—C14	−71 (2)
C5—C4—C3—C2	0.1 (4)	N3—C17—C18—C19	0.1 (3)
C3—C4—C5—C6	−0.3 (4)	C17—C18—C19—C20	1.2 (3)
C3—C4—C5—C8	−179.7 (3)	C21—C20—C19—C18	−1.4 (3)
C4—C5—C6—C7	0.2 (4)	C22—C20—C19—C18	−175.1 (2)
C8—C5—C6—C7	179.7 (3)	N3—C21—C20—C19	0.4 (3)
C2—C7—C6—C5	0.0 (4)	N3—C21—C20—C22	173.90 (17)
O3—C9—C10—C11	177.3 (2)	O5—C22—C20—C19	48.0 (3)
O3—C9—C10—C15	−3.7 (3)	O5—C22—C20—C21	−125.4 (2)
O4—C9—C10—C11	−3.3 (3)	N4—C22—C20—C19	−133.0 (2)
O4—C9—C10—C15	175.6 (2)	N4—C22—C20—C21	53.6 (3)

Symmetry codes: (i) −*x*+1/2, *y*−1/2, −*z*+1/2; (ii) −*x*+1/2, *y*+1/2, −*z*+1/2.

### Hydrogen-bond geometry (Å, º)

**Table:**

*D*—H···*A*	*D*—H	H···*A*	*D*···*A*	*D*—H···*A*
O6—H61···O3^iii^	0.81 (2)	1.83 (2)	2.630 (2)	171 (3)
O6—H62···O1^iii^	0.79 (2)	1.90 (2)	2.673 (2)	166 (3)
C18—H18···O2^iv^	0.93	2.55	3.460 (3)	166
C21—H21···O5^i^	0.93	2.45	3.054 (3)	123
C23—H23*B*···O6^ii^	0.97	2.32	3.208 (3)	152

Symmetry codes: (i) −*x*+1/2, *y*−1/2, −*z*+1/2; (ii) −*x*+1/2, *y*+1/2, −*z*+1/2; (iii) −*x*, −*y*, −*z*; (iv) *x*, *y*+1, *z*.
